# How Do Patients' Fear Prediction and Fear Experience Impact Exposure-Based Therapy for Panic Disorder With Agoraphobia? A Comprehensive Analysis of Fear Prediction

**DOI:** 10.1155/da/6963860

**Published:** 2025-06-18

**Authors:** Marina Hilleke, Thomas Lang, Sylvia Helbig-Lang, Georg W. Alpers, Volker Arolt, Jürgen Deckert, Thomas Fydrich, Alfons O. Hamm, Tilo Kircher, Jan Richter, Andreas Ströhle, Hans-Ulrich Wittchen, Alexander L. Gerlach

**Affiliations:** ^1^Department of Clinical Psychology and Psychotherapy, University of Cologne, Cologne, Germany; ^2^Christoph-Dornier-Foundation for Clinical Psychology, Bremen, Germany; ^3^School of Business, Social and Decision Sciences, Constructor University Bremen, Bremen, Germany; ^4^Department of Psychology and Psychotherapy, University of Hamburg, Hamburg, Germany; ^5^Department of Psychology, School of Social Sciences and Otto Selz Institute, University of Mannheim, Mannheim, Germany; ^6^Institute for Translational Psychiatry, Center for Psychosocial Medicine, University of Münster, Münster, Germany; ^7^Department of Psychiatry, Psychosomatics, and Psychotherapy, University of Würzburg, Würzburg, Germany; ^8^Department of Psychology, Humboldt-Universität of Berlin, Berlin, Germany; ^9^Department of Biological and Clinical Psychology, University of Greifswald, Greifswald, Germany; ^10^Department of Psychiatry and Psychotherapy, Center for Mind, Brain and Behavior-CMBB, Philipps-University Marburg, Marburg, Germany; ^11^Department of Experimental Psychopathology, Institute for Psychology, University of Hildesheim, Hildesheim, Germany; ^12^Department of Psychiatry and Psychotherapy, Charité-Universitätsmedizin Berlin, Campus Charité Mitte, Berlin, Germany; ^13^Clinical Psychology and Psychotherapy RG, Department of Psychiatry and Psychotherapy, Ludwig-Maximilians-Universität München, München, Germany

## Abstract

Expectancy violation has been proposed as a potential core mechanism of action in psychotherapy, particularly in exposure therapy for anxiety disorders. However, various relevant expectations have been discussed, and empirical studies examining their significance are still scarce. This study aimed to investigate one specific form of expectancy violation, based on Rachman's (1994) match-mismatch model, specifically by comparing expected and experienced fear and examining their relationship to safety behaviour during exposure in vivo in 268 patients meeting DSM-IV criteria for panic disorder with agoraphobia. Participants underwent exposure to a highly controlled manual-based cognitive behaviour therapy in a randomised multicenter psychotherapy study. Participants tended to overpredict fear during exposure. Both expected and experienced fear significantly decreased over the course of repeated exposure exercises, while prediction (in)accuracy (difference between expected and experienced fear) remained stable. The decrease in expected fear over time was a strong predictor of treatment outcomes for the Bodily Sensations Questionnaire (BSQ) and Panic and Agoraphobia Scale (PAS) at post. Even more, the reduction in expected fear was a significant predictor of treatment success across all outcome measures in the follow-up assessment. These findings suggest that violating excessive fear expectancies is not a necessary condition for symptom reduction during exposure therapy.

## 1. Introduction

Cognitive behaviour therapy is an effective treatment for panic disorder with and without agoraphobia [1–3]. Exposure in vivo leads to a significant reduction of anxiety symptoms in panic disorder and agoraphobia [3–5]. Substantial evidence from experimental and preclinical research indicates that repeated exposure to feared stimuli is a prerequisite for long-term fear reduction (e.g., [6]). However, the fundamental mechanisms underlying these treatment effects have not yet been fully elucidated.

One possible mechanism is habituation, which describes the reduction of a response to a stimulus when the stimulus is repeatedly presented [7]. It is a well-studied mechanism, but its explanatory value is surprisingly limited [8], especially when considering anxiety reduction in exposure therapy [9], fear recurrence (e.g., [10–13]) and the limitations of predictions in long-term outcomes of extinction studies [14–19]. This suggests that habituation alone does not fully account for treatment effects of exposure therapy [20, 21], raising the question of whether another primary mechanism plays a central role.

Recent theories have proposed expectancy violation as a fundamental mechanism in exposure therapy. Both inhibitory and extinction learning theories are concerned with expectancy violation. The inhibitory learning model focuses on forming new safety associations that inhibit the original fear response. This process is driven by expectancy violation: When patients learn during exposure therapy that their feared outcome (such as the occurrence of a heart attack during a panic attack on a bus) does not occur, a new memory trace is formed. This trace inhibits the retrieval of the original fear memory and its associated fear response (for more information see [8, 21–23]). A persistent challenge in inhibitory learning is the return of fear in novel contexts or over time, referred to as contextual renewal and spontaneous recovery, respectively (for more information see [21]). While inhibitory learning emphasises generating new memories to inhibit old fear responses, extinction learning aims to reduce the conditioned fear response itself [24–26]. At least partially, extinction learning considers pathological fear as a conditioned response that arises from the pairing of a conditioned stimulus with an unconditioned threat. For example, in panic disorder, panic attacks are thought to result from interoceptive stimuli that have been associated with threat [27]. Craske et al. [21] suggested that a significant reduction of fear in exposure therapy should occur if there is a mismatch of expectations of an aversive event and the subsequent experience that the aversive event does not transpire. Consequently, patients should experience the feared situation as less aversive than expected and repeat this experience. Thus, both inhibitory and extinction learning suggest that expectancy violation plays a central role in fear reduction. However, an open question remains: to what extent does expectancy violation contribute to fear reduction in exposure therapy and how might different types of expectations influence this process?

A recent study conducted by Pittig et al. [28] investigated the relationship between expectancy and therapy outcome. More precisely, the study examined the violation and change of threat beliefs in a sample comprising various anxiety disorders during exposure. Threat beliefs refer to the extent to which individual endorse catastrophic thoughts, such as the belief that they might die from a panic attack due to a heart attack which in turn results in strong fears. The study found that expectancy violation did not predict therapy success per se, but served as an important prerequisite for expectancy change, which, in turn, was associated with a better therapy outcome. In particular, the learning rate emerged the strongest predictor.

In contrast, the match–mismatch model by Rachman [29] emphasises fear prediction rather than the expectation of a specific catastrophic outcome. It focuses on the predicted intensity of fear before confronted with a threatening stimulus and compares it to the actual experienced fear during exposure. Central to the match–mismatch model is the assessment of both predicted and experienced fear, as well as the discrepancy between them. This approach enables an understanding of anxiety in which correcting inflated fear expectations could play a crucial role. The match–mismatch model [29] assumes that fearful individuals tend to overpredict the fear they will experience in a threatening situation. Indeed, several studies have provided empirical support for the match–mismatch model of fear in panic disorder and agoraphobia [30–33]. Based on this model, a perceived mismatch between expected and experienced fear may constitute a fundamental form of expectancy violation and thus contribute to exposure therapy outcomes. Nonetheless, it remains unclear whether increased accuracy in fear prediction can forecast treatment outcomes.

Numerous studies have shown that the use of safety behaviours during exposure exercises negatively impacts the effectiveness of exposure therapy [34–37]. Furthermore, Craske et al. [21] suggested that distraction interferes with expectancy change and may thus hinder new learning. Several studies have reported a strong correlation between fear prediction, experienced fear and avoidance behaviour [38–41]. Consequently, as anxious people tend to overpredict their fear [42], safety behaviours may reinforce and maintain this overprediction bias by preventing the correction of anticipated fear. This overprediction bias might contribute to the development of anticipatory anxiety and avoidance behaviour, which in turn impedes the disconfirmation of feared outcomes (for further information, compare Rachman [29] or Hilleke et al. [42]).

This study examines the role of expected fear within the framework of Rachman's [29] match–mismatch model during exposure sessions in patients with panic disorder with agoraphobia. To date, no research has investigated whether fear expectancies become more accurate with an increasing number of exposure trials and whether this is significantly related to therapy outcomes in panic disorder and agoraphobia. Given that individuals with panic disorder tend to systematically overestimate their fear [42], it can be assumed that improved fear assessment reduces misperceptions, thereby facilitating the refutation of irrational beliefs, which may in turn positively affect therapy outcomes. Furthermore, accurate fear assessment may reduce avoidance and safety behaviour and encourage individuals to confront anxiety-provoking situations more confidently. Due to the design of the study, this investigation focuses exclusively on safety behaviour. Specifically, this study investigates the impact of safety behaviour on the violation of expected fear and on the course of anxiety. Thus, this study examines the overprediction of fear and the role of expectancy violation in patients diagnosed with panic disorder with agoraphobia during in vivo exposure. Additionally, it explores the effect of safety behaviour based on the following hypotheses:

1. Patients with panic disorder with agoraphobia overpredict the fear they will experience in a threatening situation in their first exposure session.
2. The predictions of fear of patients with panic disorder with agoraphobia will become more accurate with a growing number of repeated exposure trials during treatment.
3. Improvement of fear predictions is associated with better treatment outcomes.
4. Overprediction of fear is associated with safety behaviour used during exposure sessions.

## 2. Methods

### 2.1. Study Design and Procedures

Data were collected in the clinical multicenter trial on mechanisms of action in CBT (MAC) [43]. Eight German study centres (Aachen, Berlin Adlershof, Berlin-Charite, Bremen, Dresden, Greifswald, Münster and Würzburg) participated in the trial. To be included, participants had to meet DSM-IV-TR diagnostic criteria for panic disorder with agoraphobia. Diagnoses were established using a standardised clinical diagnostic interview, an adapted version of the Composite International Diagnostic Interview (DIA-X) [44]. In addition, a score of at least ≥18 on the Hamilton Anxiety Scale (SIGH-A) structured interview and a score of at least >3 on the Global Impressions Scale (CGI) was required. Participants had to be between 18 and 65 years of age. Individuals with psychotic or bipolar disorder, alcohol dependence, alcohol abuse, dependence on benzodiazepines or other psychoactive substances, suicidal intent, medical contraindications (e.g., severe cardiovascular diseases) or those meeting the diagnostic criteria for borderline personality disorder were excluded. Patients who were unable to participate in the treatment due to organisational or time constraints were also excluded. Additional exclusion criteria included receiving other psychotherapeutic or psychopharmacological treatments (for more information, see [43, 45]).

### 2.2. Treatment

The treatment consisted of 12 sessions of exposure-based CBT [46], conducted twice weekly. The two active conditions were either therapist-guided or self-guided exposure. In the self-guided group, exposure sessions were prepared in advance with the therapist. In both groups, patients were required to complete additional exposure exercises as homework.

Treatment consisted of the following elements: Sessions 1–3: psychoeducation. Session 4 and 5: Explanation of the therapy rationale and interoceptive exposure. In Sessions 6–8 and 10–11 execution and/or discussion of in vivo exposures. After Sessions 6–11, participants were instructed to repeat the discussed or conducted exposure exercises on their own as homework. In Sessions 9 and 12, there was a review of the course of therapy and an analysis of changes in anticipatory anxiety throughout treatment. In Session 12, there was also a discussion of therapeutic gains and relapse prevention. Two final booster sessions included individual plans for exposure and relapse prevention. The measurement and analysis of expectancy violation are described below.

### 2.3. Therapists

The therapists administering the treatment had completed their psychology degrees and were undergoing advanced CBT training. They attended a 3-day workshop on the manualized therapy and assessment procedures, which included role-play of core treatment components. Following this workshop, therapists were required to submit a recorded role-play, which was evaluated by experts. Only those who successfully passed the role-play were allowed to treat study patients. To demonstrate their proficiency, therapists submitted videotapes and example ratings. Furthermore, weekly supervision sessions were conducted to ensure adherence to the treatment protocol (for more information, see [45]).

### 2.4. Participants

The study involved 369 participants. Three hundred one of them were randomly assigned to one of two active treatment conditions, while 68 participants were placed on a wait list control group. For the current analysis, only participants who completed at least one exposure session were included. Accordingly, 33 patients who dropped out prior to the first exposure session were excluded, resulting in a final sample of 268 patients. The mean age of these 268 participants was *M* = 35.3 (*SD* = 10.5) and 75% were female. Seventy-six percent of the participants had at least one comorbid diagnosis. The most common comorbidities were specific phobias (*n* = 127), depressive disorders (*n* = 104), somatoform disorders (*n* = 60), social phobias (*n* = 56) and substance use disorders (*n* = 49).

### 2.5. Assessments

The outcome measures required for the current study are described below. Refer to Gloster et al. [43] for a complete assessment list.

#### 2.5.1. Agoraphobic Cognitions Questionnaire (ACQ) [47]

The questionnaire assesses the frequency of anxiety-related cognitions on a 5-point Likert-type scale. The ACQ's reliability is considered sufficient (*α* = 0.80, *r* = 0.79) [47].

#### 2.5.2. Bodily Sensations Questionnaire (BSQ) [47]

The BSQ includes 17 body sensations typically associated with autonomic arousal. Respondents are asked to rate how much anxiety each of these symptoms causes on a 5-point Likert-type scale. The total score reflects the anxiety associated with the bodily symptoms. See [47] for internal consistency (*α* = 0.87) and retest-reliability (*r* = 0.67).

#### 2.5.3. Mobility Inventory (MI) [48]

The MI measures agoraphobic avoidance behaviour in situations with and without company (range 1–5). These two scales assess the frequency of avoidance behaviour in these situations. The MI has an internal consistency of *α* = 0.94 and a reliability of *r* = 0.90 [48].

#### 2.5.4. Panic and Agoraphobia Scale (PAS) [49]

The PAS is a self-report questionnaire specifically designed to monitor changes during treatment of panic disorder with agoraphobia. The 13 items can be assigned to five subscales: panic attacks, agoraphobic avoidance, anticipatory anxiety, disability and health worries. The total score reflects the severity of panic disorder with agoraphobia. According to Hoyer et al. [50], the PAS has a retest reliability of *r* = 0.78 and an internal consistency of *α* = 0.85.

#### 2.5.5. Exposure Protocol and Fear Expectancy Assessment

All exposures in the therapy process (both in therapy and as homework) were documented on a specially prepared protocol sheet. These protocols included questions about the specific exposure session (length of exposure, various questions concerning anxiety and elements of anxiety, safety behaviour, the willingness to perform the exercise, etc.). This protocol also recorded the level of anxiety that the patient expected before exposure: ‘What will be the maximum level of anxiety in the situation?'. The Likert-type scales ranged from 0 = no fear to 10 = extreme fear. The experienced anxiety was measured after exposure: ‘What was the maximum level of anxiety during the exercise?'. The answer was again rated on a 0–10 Likert-type scale (0 = no fear-10 = extreme fear). After each exposure, patients recorded their fear-intensity using a Likert-type scale, recorded the course of their subjectively experienced anxiety using a drawn line, documented any safety behaviours they used and provided personal reflections on the exercise. Therapists separately recorded all safety behaviours the patients employed during the exercise.

#### 2.5.6. Safety Behaviour During Exposure

Safety behaviour was assessed using two different questions in the protocol sheet. All exposures were documented: ‘Did anything in the situation help you to cope with the fear or anxiety?' and ‘During the exercise, did you try to control the fear or any symptoms?'. The patients could state ‘yes', ‘partly', or ‘no'. In free text, they could indicate what kind of safety behaviour they had used. In the next step, two independent raters compared the data of the protocol sheets. These two independent raters assigned the information as either 1 (safety behaviour present) or 0 (no safety behaviour present). The raters' agreement was high and significant (Cohens Kappa = 0.92). If the person completely avoided exposure, the protocol was not completed. These patients continued outpatient treatment but were excluded from the study.

### 2.6. Data Analyses

#### 2.6.1. Outcome Evaluation

The treatment outcome was defined a priori as differences in scores between the baseline and post/follow-up assessment scores for the primary outcomes MI scales (accompanied and unaccompanied) and PAS. The secondary outcomes were ACQ and BSQ. A higher change score indicated a more substantial improvement in symptoms and, consequently, a better treatment outcome.

#### 2.6.2. Process Measures

The information of the exposure protocol was used to create indicators of emotional processing. Expected fear and experienced fear were both directly assessed. The difference between the two scores was calculated (expected fear–experienced fear).

### 2.7. Analytic Strategy

Given that no differences were observed with regard to the outcome measures (ACQ, BSQ and MI) between the therapist guided and patients led group (for more details see [46]), for the present analyses, treatment groups were combined.

#### 2.7.1. Hypothesis 1

Based on the calculations of Rachman and Lopatka [51, 52], the overprediction bias of fearful subjects in a threatening situation during the first exposure (first hypothesis) was evaluated using a chi-square test. A deviation of up to 1 was allowed to be classified as a match. If the expected anxiety was higher than the experienced anxiety by more than 1, it was considered an overprediction. If the expected anxiety was lower than the experienced anxiety by more than 1, it was considered an underprediction. The calculation was conducted following Telch et al. [53] or van Hout and Emmelkamp [31].

#### 2.7.2. Hypotheses 2–4

**Figure 1:**
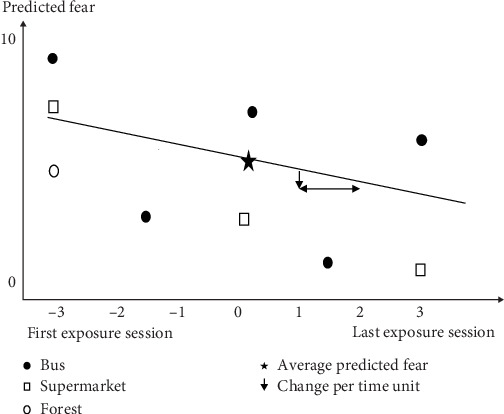
Demonstration of the analytic strategy of the data of one person. To standardise the different number of exposures per patient, a time coding system was used per exposure with −3 as the time code for the first exercise session and +3 for the last repetition.

Data from 3.376 exposure exercises were analysed using MPlus (version 6.1). For each person, sessions with exposure to six different situations were analysed (standardised exercises: bus, supermarket, forest, repetition of one of these standardised exercises and two individual situations). Patients differed in the number of repetitions of these exercises (including homework tasks). To standardise the data set despite these differences, a time coding system was used per exposure with −3 as the time code for the first exercise session and +3 for the last repetition. The first session was coded with −3 and the last with +3. Time Point 0 is used to indicate average values (Figure 1). For an example of a dataset of one person and three of the exposure situations (bus, supermarket and forest; Figure 1). As can be seen, this person entered bus situation five times. Consequently, the time coding for the first session is −3 and the last session is +3. The other three exposure sessions for the bus were equally spaced in between. The exposure to the supermarket was repeated three times. Here, the first session is coded −3 and the last session +3. The second session is equally spaced in between, here coded as 0. The exposure to a forest was entered only once. The time constant of the situation entered only once is −3.

Using this coding system, the information on exposures performed by a person was summarised. For each person, the following indicators of emotional processing were calculated: expected fear, experienced fear, the difference between expected and experienced fear and safety behaviour.

Data on predicted fear was summarised in two parameters: The asterisk in Figure 1 indicates the predicted fear at a time Code 0 (average predicted fear). The downward-pointing arrow indicates the change in predicted fear per unit of time and represents the indicator for the ongoing reduction in an exposure situation. The same procedure was applied to experienced fear. A negative value for expected and experienced fear over time represented a decrease in expected and experienced fear.

The difference between expected and experienced fear could be between −10 and 10. Positive values meant that the expected fear was higher than the experienced fear.

All analyses were conducted using the multilevel modelling approach implemented in Mplus (www.statmodel.com). The base model was a bivariate multilevel model (with the dependent variables ‘expected fear', ‘experienced fear', and the ‘difference between expected and experienced fear'). The analyses used to evaluate the research hypotheses are described in detail below.

Hypothesis 2 examined the relationship between the different kinds of fears. Estimates for expected fear, experienced fear and the difference between the two fear variants were calculated. The analysis was executed using the Mplus command ESTIMATOR = BAYES.

Hypothesis 3 analysed the relationship between different types of fears and treatment outcomes. We tested whether the treatment outcome could be predicted by each indicator (expected fear, experienced fear and difference between expected and experienced fear). Primary outcome measures were calculated as the change from between pretreatment and post-treatment scores and between pretreatment and follow-up scores. These analyses was performed with the Mplus command ESTIMATOR = ML.

In Hypothesis 4, regression analyses were used to examine the relationship between safety behaviour and expected fear, experienced fear and the difference between expected and experienced fear. This analysis was also conducted using the Mplus command ESTIMATOR = BAYES.

## 3. Results

### 3.1. Treatment Outcome

The outcome measures showed significant reductions from pre-assessment to post-assessment and from preassessment to follow-up assessment. The results were as follows: ACQ: Pre = 2.16 (0.55), Post = 1.64 (0.46), FU = 1.49 (0.46), *η*^2^ = 0.57; BSQ: Pre = 2.81 (0.72), Post = 2.06 (0.70), FU = 1.94 (0.72), *η*^2^ = 0.51; MIacc (MI, subscale accompanied): Pre = 2.22 (0.72), Post = 1.50 (0.63), FU = 1.35 (0.58), *η*^2^ = 0.58; MIunac (MI, subscale unaccompanied): Pre = 2.95 (0.81), Post = 1.98 (0.86), FU = 1.66 (0.84), *η*^2^ = 0.64; PAS: Pre = 27.72 (9.76), Post = 14.84 (9.20), FU = 10.51 (9.01), *η*^2^ = 0.74. For more information, see [43, 45].

### 3.2. Number of In-Situ Exposure Exercises

The number of exposure exercises completed by participants ranged from 1 to 44, with a mean of 13.4 (SD = 8.3). The majority of patients (84%) underwent between 10 and 30 exposure exercises during treatment: 9% (*n* = 24) did up to five and 3% (*n* = 8) did more than 30 exposure exercises.

### 3.3. Expected Fear, Experienced Fear and the Difference Between Them

In most of the 44 trials conducted per participant (Table A1), there was a significant difference between expected and experienced fear. Exposure situations included taking the bus, going to a supermarket, going to a forest and two individually defined exposure situations. All participants followed the same sequence of exposures (Table A1).

### 3.4. Overprediction Bias (Hypothesis 1)

Out of 241 valid trials of the first exposure situation (27 invalid trials due to missing indication in the protocols) in 97 exposures, expected fear matched experienced fear (36.2%). Patients overpredicted fear in 127 (47.4%) cases and underpredicted fear in 17 cases (6.3%). This difference between overpredictors, underpredictors and matchers was significant, as calculated with a chi-square test (*X*^2^ = 80.50, *p*  < 0.01). The first hypothesis was confirmed.

### 3.5. Changes in the Accuracy of Predictions (Hypothesis 2)

As shown in Table 1, the expected fear was, on average, 6.07 and the experienced fear was 4.70. The change in expected fear over time was −1.38. More precisely the change in predicted fear over time for an average person between the time point −3 and +3 is −0.23. Since −0.23 represents the change in predicted fear for one time unit it needs to be multiplied by 6: 6 × −0.23 = −1.38 (see analytic strategies). The change in experienced fear over time was −1.26 (6 times −0.21). Although both expected and experienced fear decreased significantly over time, the prediction accuracy, remained stable, with a difference score of −0.12 (6 times −0.02). The second hypothesis, therefore, has to be rejected.

The analyses were repeated for each exposure situation (bus, department store and forest). In these analyses, there was also no significant change in difference scores between expected and experienced fear over time; only a trend in the situations bus, forest and the second individual situation toward improvement in predictions was observed (Tables A2–A6).

The correlations suggest the following interpretations: The higher the levels of both expected and experienced fear, the smaller the reduction in the difference between them over time. Additionally, a slower decrease in expected fear corresponds to a smaller overall reduction in this difference.

### 3.6. Relationship Between Expected Fear, Experienced Fear and Accuracy of Prediction With Therapy Outcome (Hypothesis 3)

Table 2 summarises the results of the regression analyses using the outcome measures as dependent variables. Expected fear and the difference between expected and experienced fear were taken as predictors. As shown in the pre–post assessment, the reduction of the expected fear over time was a significant predictor for the outcome measures BSQ and PAS. This means the more substantial the reduction in expected fear, the better the therapeutic outcome. The change in the difference between expected fear and experienced fear over time was not significantly related to any outcome measure.

When comparing changes from pre to follow-up with respect to expected fear over time, the change of expected fear can be identified as a significant predictor of treatment success in all outcome measures. The change in difference scores of expected and experienced fear over time was not significantly related to therapeutic outcome (Table 3). The Appendix A includes some further exploratory calculations with regard to experienced fear in relation to the therapy outcome (Tables A7 and A8). Even there, it can be observed that the experienced fear over time, both from pre to post and from pre to follow-up, significantly influenced the treatment outcome except for the MI accompanied.

### 3.7. Relationship Between Expected Fear, Experienced Fear and the Difference Between Expected Fear and Experienced Fear With Safety Behaviour (Hypothesis 4)

Table 4 shows no significant association between safety behaviour and expected fear, experienced fear and the difference between expected and experienced fear. Therefore, the fourth hypothesis has also to be rejected.

## 4. Discussion

The present study investigated the impact of reported discrepancies between expected and experienced fear during fear exposure exercises (i.e., fear expectancy violation) on treatment outcome. Additionally, the role of safety behaviours during exposure in vivo in panic disorder with agoraphobia regarding this relationship. As hypothesised, patients overestimated the fear they would experience in a threatening situation during their first exposure session. However, our core finding is that the predictions were not more accurate with the number of exposure trials during treatment. In other words, the accuracy or change in accuracy of these predictions was not correlated with treatment outcomes. However, the change in expected and experienced fear was significantly associated with certain treatment outcomes. The impact on expected fear was significant of BSQ and PAS at postassessment, while all outcome measures were significant at follow-up. In contrast, experienced fear did not show significance for MIacc at either postassessment or follow-up. These findings suggest that reductions in both expected and experienced fear contribute to improved therapy outcomes, with greater decreases across exposures leading to more favourable results. Finally, there was no association between the use of safety behaviour and the accuracy of the predictions.

The supporting findings of the overprediction bias which was investigated in the first hypothesis align with previous results ([29, 54]; for a summary, see [42]). Anxious individuals frequently overestimate the level of anxiety they will experience when confronted with a fear-provoking situation. This phenomenon appears to be specific to anxious individuals and may partially explain the maintenance of anxiety disorders. One explanation for the overprediction bias is that individuals with high anxiety levels tend to exhibit an attentional bias towards threats [55]. This bias could then possibly lead to overestimation and increased anxiety.

In the second hypothesis, it emerged that the fear predictions were not more accurate with the number of exposure trials during treatment. The overprediction bias persisted over the course of repeated exposure exercises. Please note that the exposure situations changed: first the bus, then, the supermarket, then, the forest, then, two individual situations. Even when considering only changes within situations or within persons (e.g., only exposure sessions in the bus situation), no significant change in terms of accuracy of the prediction of anxiety could be found. Interestingly, higher levels of both expected and experienced fear were associated to smaller reductions in the difference between them over time. A slower decline in expected fear corresponded to a less pronounced decrease in this difference. This suggests a relationship between expected fear and the accuracy of fear prediction, warranting further investigation in future studies.

Findings from prior studies regarding prediction accuracy are mixed (see, e.g., [30, 51–53, 56]). For example, Telch et al. [53] found an increase in the number of patients who were able to predict their anxiety (‘matchers') accurately. However, even in that study, some patients continued to overestimate their anxiety despite overall reductions in both expected and experienced fear. In contrast and line with our results, van Hout and Emmelkamp [31] did not find an increase in accuracy across treatment blocks in fear predictions, that is, the mean difference between predicted and real fear showed no significant change.

The inconsistent findings in former research and the present study might be partly explained by methodological differences. Previous studies used typically *t*-tests or ANOVA analyses to assess group differences, which could potentially obscure individual differences. This paper used multilevel models so that individual trajectories can be taken into account.

The varying number of patients who overestimated, correctly estimated or underestimated and the level of overestimation in the different samples could also partially explain different results. In samples with higher levels of initial overestimation, the corrective experience may be more likely to align with expected and experienced anxiety. As a result, the probability that expected and experienced anxiety may converge is greater.

In line with inhibitory learning theory, Craske et al. [8] emphasised that the critical factor may not be the specific anticipation of fear in a particular situation. Craske et al. [8] proposed that it is an individual's expectation regarding their worst-case scenarios and the perceived likelihood of those threat scenarios occurring. The focus would shift from the difference between the specific expected and experienced fear during the exposure situation (i.e., accuracy) to whether the individual effectively rectifies their mistaken assumptions about the perils associated with that situation. It would also have to be checked whether the learning experience is transferred from one situation to another. It is unclear whether every form of expectation violation leads to relevant learning experiences.

The results regarding the third hypothesis indicated that the discrepancy between expected and experienced fear had no impact on treatment outcomes. Only the change in expected fear over time showed significant correlations with therapy outcomes. Changes from baseline to follow-up assessment were more related to therapy outcome than changes from pre- to postassessment. These findings suggest that the degree of mismatch between experienced and expected fear, as posited by Rachman's match–mismatch model, may not be critical for symptom reduction in exposure therapy in panic disorder with agoraphobia. Rather, a general reduction in fear predictions appears to be important. Importantly, even within a time-limited treatment, individuals with anxiety disorders may persistently overestimate their fear, despite a decrease in fear levels across exposures.

Rief et al. [57] identified expectations as a central maintaining mechanism in mental disorders such as panic disorder and agoraphobia. Different types of expectations, such as threat expectations and fear expectations, may differentially influence therapy outcomes. Pittig et al. [28] found that a higher learning rate and greater changes in threat expectancy levels were associated with a more effective treatment outcome than the expectancy violation itself. In this regard, threat expectancy emerges as an especially important factor.

The present findings support the habituation model, which posits that greater fear reduction during exposure enhances treatment outcomes. Maybe multiple mechanisms of action help explain the effect of exposure therapy, depending on whether patients are more afraid of anxiety symptoms or anxious thoughts. Furthermore, it should be taken into account that many patients fear the panic symptoms themselves. Therefore, future research should not only consider catastrophic events but also catastrophic internal expectations. Given the limited data available on expectancy violation and therapy outcomes, further investigation is clearly warranted.

There was no significant correlation between safety behaviour and prediction accuracy which was investigated in hypothesis four. In contrast to our findings, the prevailing evidence largely suggests that most forms of safety behaviours diminish the efficacy of exposure-based treatments [37]. For example, a study by Salkovskis et al. [35] indicates that the use of safety behaviours is associated with a smaller reduction in catastrophic beliefs as well as in anxiety. In our study, safety behaviours do not seem to influence prediction accuracy, though this should be examined in more detail for the following reasons. First, our measurement only provided a broad safety behaviour estimate, making only a rather general distinction between its utilisation and non-utilisation. Future studies will benefit from assessing safety behaviour more thoroughly. Moreover, as mentioned before different types of expectation could influence safety behaviour. For example, it may be important to differentiate between the expectation of a catastrophic event (inhibitory learning) or the expectation of intense fear (match–mismatch model). Anticipating a devastating event may lead to an increased fear response, triggering heightened safety behaviour. Consequently, it seems important to differentiate between safety behaviours aimed at reducing anxiety (e.g., distraction) and those intended to directly prevent a harmful outcome (e.g., monitoring people coming and going to avoid an attack).

What implications do the results have for therapeutic practice? The present results revealed that patients consistently overestimate their fear during exposure therapy. Possibly, predictions and overestimation of fear should be addressed more directly in treatment. The effects of overestimation should be explained in more detail and should explicitly be documented before and after exposure therapy. It might be beneficial after exposure to highlight expectancy violations and thus maximise the representation of expectation violation effects.

Our findings suggest that heightened expected and experienced fear appear to impair prediction accuracy. Applying this information to exposure therapy highlights the importance of ensuring that fear activation is present within the therapeutic framework. On the other hand, excessively high fear activation may be detrimental on the therapeutic outcome. Achieving a moderate level of fear may optimise treatment outcome. This aligns with Foa and Kozak [58] recommendation that fear activation beside within-session reduction and between-session fear reduction is crucial in therapy. Meuret et al. [59] also showed that a moderate anxiety response is most beneficial in exposure.

Overprediction bias may also lead some patients to avoid or drop out of exposure therapy. Approaches like desensitisation [60] or graduated exposure (e.g., [61]) can help by only gradually increasing challenging levels and following a fear hierarchy. Using retrieval cues in new contexts may also help [62–64]. Care should be taken to ensure that these cues do not inadvertently function as safety behaviours [65]. Despite this risk, such methods may encourage patients to engage in exposure therapy. Context changes during exposure are also recommended [66].

Although safety behaviour was not related to fear and overprediction in our study, we suggest encouraging patients to abstain from safety behaviours (compare [34, 37]). Arguably, there is at least a chance that fear activation might be more successful if safety behaviours are restricted (e.g., [67]).

Several limitations of this study should be acknowledged. The level of anxiety was assessed solely via self-report. Incorporating physiological or behavioural measures (e.g., [68]) could provide a more comprehensive assessment. In addition, the time spent in each exposure situation was limited due to time constraints within the treatment study. It, thus, remains unknown if more exposures in each one of the situations would have given different results. As seen in Table A1, the individuals in this sample completed different numbers of exposures across the different situations. Thus, some individuals are included that confronted only one situation. Perhaps the results would be different if all participants had exposure assignments over the same number of repetitions. Furthermore, the levels of anxiety differ substantially in the diverse situations across individuals. Most importantly, due to the first three exposure situations being prescribed, some situations may have elicited only little or no anxiety in some participants. This may have influenced our measures in terms of accuracy. If the fearful situation triggers higher levels of fear, it may also lead to a more substantial violation of expectations. Thus, the design of the present treatment study may have negatively impacted our results.

## 5. Conclusion

The main conclusion of the present study is that patients with panic disorder with agoraphobia indeed overestimate their anxiety. Prediction accuracy did not improve with treatment. Moreover, the accuracy of the fear predictions was not correlated with therapy outcomes, whereas the report of fear reductions was. Surprisingly, safety behaviours also had no significant impact on fear reports. These findings suggest that violating fear expectancies as derived from Rachman's match–mismatch model, may not be critical for symptom reduction during exposure therapy. In future studies, the model should be tested in additional clinical samples presenting various anxiety disorders. In subsequent studies, expectation violations regarding the assumption of specific, individually anticipated catastrophic events should be examined as possible specific mechanisms of exposure therapy. Additionally, it seems essential for further research to separate the different facets of expectancy as prediction of catastrophic events, prediction of the most feared bodily or mental symptom and predictions of new exposure to the same situations after exposure. Further investigation is needed to determine whether different facets of expectancies influence various types of safety behaviours. Future studies should also address the key question: Why do patients with panic disorder and agoraphobia continue to overestimate fear despite experiencing anxiety reductions and which factors maintain this misperception?

## Figures and Tables

**Table 1:** Estimates and variances of expected fear, experienced fear and difference of expected and experienced fear and their intercorrelations (between brackets significances of the corresponding covariances).

Estimates (variances)			Intercorrelations					
Estimate		*p*	Expected fear	Experienced fear	Diff	F1Time	F2Time	DTime
Expected fear	**6.07 (4.01)**	**<0.01**	—	—	—	—	—	—
Experienced fear	**4.69 (4.23)**	**<0.01**	**0.90 (<0.01)**	—	—	—	—	—
Diff	**1.47 (0.82)**	**<0.01**	**0.15 (0.03)**	**−0.29 (<0.01)**	—	—	—	—
F1Time	**−0.23 (0.01)**	**<0.01**	**0.63 (<0.01)**	**0.62 (<0.01)**	−0.04 (0.43)	—	—	—
F2Time	**−0.21 (0.01)**	**<0.01**	−0.09 (0.32)	−0.21 (0.12)	0.30 (0.07)	0.00 (0.43)	—	—
DTime	−0.02 (0.02)	n.s.	**0.46 (<0.01)**	**0.56 (<0.01)**	**−0.29 (0.03)**	**0.63 (0.01)**	**−0.78 (<0.01)**	—

*Note*: Diff, difference between expected fear and experienced fear; F1Time, expected fear over time; F2Time, experienced fear over time; DTime, difference between expected and experienced fear over time. Bold values indicate statistically significant results.

**Table 2:** Prediction of treatment outcome at postassessment of expected fear and difference between expected and experienced fear.

Outcome measures	Expected fear	Diff	F1Time	DTime	*R* ^2^
ACQ	0.33 (0.48)	0.27 (0.28)	−0.52 (0.29)	0.35 (0.38)	0.29 (0.13)
BSQ	0.47 (0.14)	0.07 (0.76)	**−0.94 (<0.01)**	0.29 (0.43)	0.60 (0.07)
MI acc	0.50 (0.26)	0.03 (0.86)	−0.60 (0.18)	0.21 (0.54)	0.30 (0.54)
MI unac	0.26 (0.59)	0.11 (0.69)	−0.45 (0.38)	0.51 (0.26)	**0.34 (0.02)**
PAS	0.49 (0.13)	0.13 (0.55)	**−0.90 (<0.01)**	0.43 (0.19)	**0.62 (0.03)**

*Note*: Beta scores (with significances of corresponding regression coefficients in brackets) and *R*^2^ (between brackets overall significance) are reported. Diff, difference between expected fear and experienced fear; DTime, difference of expected and experienced fear over time; F1Time, expected fear over time; MI acc, mobility inventory, subscale accompanied; MI unac, mobility inventory, subscale unaccompanied. Bold values indicate statistically significant results.

Abbreviations: ACQ, Agoraphobic Cognitions Questionnaire; BSQ, Bodily Sensations Questionnaire; PAS, Panic and Agoraphobia Scale.

**Table 3:** Prediction of treatment outcome at follow-up based on expected fear and the difference between expected and experienced fear.

Outcome measures	Expected fear	Diff	F1Time	DTime	*R* ^2^
ACQ	0.51 (0.11)	0.34 (0.16)	**−0.92 (0.01)**	0.38 (0.32)	**0.54 (<0.01)**
BSQ	0.47 (0.07)	0.07 (0.53)	**−0.94 (<0.01)**	0.29 (0.35)	**0.69 (0.02)**
MI acc	0.61 (0.10)	−0.05 (0.79)	**−0.94 (0.03)**	0.06 (0.85)	0.40 (0.56)
MI unac	0.54 (0.11)	0.19 (0.46)	**−0.86 (0.04)**	0.53 (0.21)	**0.53 (0.05)**
PAS	**0.67 (0.01)**	0.07 (0.72)	**−0.99 (<0.01)**	0.42 (0.15)	**0.99 (<0.01)**

*Note*. Beta scores (with significances of corresponding regression coefficients in brackets) and *R*^2^ (between brackets overall significance) are reported. MI acc, mobility inventory, subscale accompanied; MI unac, mobility inventory, subscale unaccompanied. Diff, difference between expected fear and experienced fear; F1Time, expected fear over time; DTime, difference of expected and experienced fear over time. Bold values indicate statistically significant results.

Abbreviations: ACQ, Agoraphobic Cognitions Questionnaire; BSQ, Bodily Sensations Questionnaire; PAS, Panic and Agoraphobia Scale.

**Table 4:** Regression analysis of expected fear, experienced fear and the difference between expected and experienced fear by safety behaviour.

Fear estimates and time cores	Safety behaviour	*p*	STime	*p*
Expected fear	−0.46	0.19	—	—
Experienced fear	0.75	0.07	—	—
Diff	0.65	0.10	—	—
F1Time	1.28	0.25	−0.04	0.45
F2Time	−0.18	0.46	0.08	0.40
DTime	−0.87	0.33	0.01	0.49

*Note*: Diff, difference between expected fear and experienced fear; F1Time, expected fear over time; F2Time, experienced fear over time; DTime, difference between expected and fear experienced over time; STime, safety behaviour over time.

**Table 5:** Expected fear, experienced fear and the difference between experienced and expected fear across all types of trials.

Exposure trial	*N*	Expected fear *M* (SD)	Experienced fear *M* (SD)	*T*	*p*	Difference *M* (SD)
1 bus	241	7.70 (2.40)	5.80 (2.97)	11.60	<0.001	1.89 (2.53)
2 bus	179	5.78 (2.80)	4.29 (2.94)	8.12	<0.001	1.49 (2.45)
3 bus	38	5.57 (2.61)	3.71 (2.61)	5.86	<0.001	1.86 (1.95)
4 bus	12	6.58 (2.44)	4.58 (3.00)	4.23	0.001	2.00 (1.64)
5 bus hw	124	6.22 (3.07)	4.77 (3.08)	8.74	<0.001	1.45 (1.85)
6 bus hw	90	5.48 (2.80)	3.78 (2.80)	8.64	<0.001	1.70 (1.87)
7 bus hw	29	6.66 (2.92)	4.98 (3.07)	4.72	<0.001	1.67 (1.91)
8 bus hw	10	5.20 (2.15)	4.40 (2.31)	1.41	—	0.80 (1.80)
9 supermarket	230	6.64 (2.91)	5.29 (3.21)	8.96	<0.001	1.35 (2.28)
10 supermarket	167	5.36 (2.99)	4.06 (3.06)	8.45	<0.001	1.30 (1.99)
11 supermarket	40	5.56 (3.22)	4.74 (3.48)	3.83	<0.001	0.83 (1.36)
12 supermarket	8	4.50 (2.83)	3.88 (3.14)	0.96	—	0.63 (1.85)
13 supermarket hw	124	5.47 (2.97)	4.19 (2.89)	7.64	<0.001	1.28 (1.87)
14 supermarket hw	87	5.03 (3.16)	3.56 (2.74)	7.62	<0.001	1.47 (1.80)
15 supermarket hw	23	5.67 (2.83)	4.35 (2.76)	4.64	<0.001	1.33 (1.37)
16 supermarket hw	7	6.57 (2.37)	4.79 (2.34)	4.75	—	1.79 (1.00)
17 forest	184	6.06 (2.90)	4.68 (3.23)	9.08	<0.001	1.39 (2.07)
18 forest	115	4.75 (3.03)	3.99 (3.09)	3.67	<0.001	0.76 (2.22)
19 forest	17	6.74 (2.44)	5.91 (2.86)	1.85	0.083	0.82 (1.84)
20 forest	3	4.33 (1.53)	4.00 (2.65)	0.38	—	0.33 (1.53)
21 forest hw	107	4.98 (3.04)	3.96 (3.15)	7.61	<0.001	1.02 (1.39)
22 forest hw	66	4.46 (3.09)	3.60 (2.81)	4.40	<0.001	0.86 (1.59)
23 forest hw	11	5.23 (3.40)	3.32 (3.21)	4.38	0.001	1.91 (1.45)
24 forest hw	5	4.80 (3.49)	2.80 (2.39)	2.39	—	2.00 (1.87)
25 repetition	124	5.70 (2.96)	4.25 (2.95)	7.45	<0.001	1.45 (2.17)
26 repetition	65	5.19 (2.92)	3.74 (2.75)	7.31	<0.001	1.45 (1.60)
27 repetition	15	5.77 (2.82)	5.17 (2.66)	1.09	0.296	0.60 (2.14)
28 repetition	9	6.56 (3.05)	6.50 (3.22)	0.32	—	0.06 (0.53)
29 first individual	211	7.30 (2.56)	5.91 (2.92)	8.83	<0.001	1.39 (2.28)
30 first individual	150	5.94 (2.77)	4.79 (2.87)	7.02	<0.001	1.16 (2.02)
31 first individual	33	6.35 (2.83)	5.17 (2.86)	3.14	0.004	1.18 (2.16)
32 first individual	7	7.43 (1.79)	5.14 (2.91)	3.03	—	2.29 (2.00)
33 first individual hw	112	6.80 (2.71)	5.08 (2.93)	9.11	<0.001	1.72 (2.00)
34 first individual hw	78	6.00 (2.77)	4.77 (2.93)	6.60	<0.001	1.24 (1.65)
35 first individual hw	25	7.00 (2.25)	5.08 (2.66)	5.05	<0.001	1.92 (1.90)
36 first individual hw	18	6.14 (2.73)	5.11 (3.00)	4.27	0.001	1.03 (1.02)
37 second individual	160	7.09 (2.64)	5.61 (3.12)	8.20	<0.001	1.48 (2.28)
38 second individual	119	5.94 (2.81)	4.68 (2.95)	7.13	<0.001	1.26 (1.92)
39 second individual	30	5.73 (2.92)	4.52 (3.11)	4.78	<0.001	1.22 (1.39)
40 second individual	5	6.40 (2.61)	5.20 (3.11)	3.21	—	1.20 (0.84)
41 second individual hw	85	6.09 (2.75)	4.61 (2.92)	6.80	<0.001	1.48 (2.00)
42 second individual hw	57	5.67 (3.17)	4.35 (3.19)	5.66	<0.001	1.32 (1.76)
43 second individual hw	21	6.19 (3.04)	4.00 (2.76)	4.26	<0.001	2.19 (2.36)
44 second individual hw	10	6.70 (3.09)	5.20 (2.81)	2.91	—	1.50 (1.63)

*Note: N* = 268; difference, difference of expected fear and experienced fear; no *p*-values are reported in trials with less than 11 participants.

Abbreviations: bus hw, bus homework; first individual hw, first individual homework; forest hw, forest homework; second individual hw, second individual homework; supermarket hw, supermarket homework.

**Table 6:** Estimates and variances of expected fear, experienced fear and difference of expected and experienced fear and their intercorrelations (between brackets significances of the corresponding covariances) in situation: bus.

Estimates (variances)			Intercorrelations					
Fear estimates and time cores	Estimate	*p*	Expected fear	Experienced fear	Diff	F1Time	F2Time	DTime
Expected fear	6.30 (4.64)	**<0.01**	—	—	—	—	—	—
Experienced fear	4.71 (5.36)	**<0.01**	**0.90 (<0.01)**	—	—	—	—	—
Diff	1.67 (1.08)	**<0.01**	0.06 (0.31)	**−0.39 (<0.01)**	—	—	—	—
F1Time	**−0.37 (0.08)**	**<0.01**	**0.52 (<0.01)**	**0.54 (<0.01)**	−0.14 (0.22)	—	—	—
F2Time	**−0.32 (0.08)**	**<0.01**	0.17 (0.11)	0.01 (0.48)	**0.35 (0.03)**	**0.68 (<0.01)**	—	—
DTime	−0.05 (0.05)	0.10	**0.40 (0.01)**	**0.62 (<0.01)**	**−0.56 (<0.01)**	**0.42 (0.04)**	−0.36 (0.07)	—

*Note*: Diff, difference between expected fear and experienced fear; DTime, difference between expected and experienced fear over time; F1Time, expected fear over time; F2Time, experienced fear over time. Bold values indicate statistically significant results.

**Table 7:** Estimates and variances of expected fear, experienced fear and difference of expected and experienced fear and their intercorrelations (between brackets significances of the corresponding covariances) in situation: supermarket.

Estimates (variances)			Intercorrelations					
Fear estimates and time cores	Estimate	*p*	Expected fear	Experienced fear	Diff	F1Time	F2Time	DTime
Expected fear	5.68 (7.38)	**<0.01**	—	—	—	—	—	—
Experienced fear	4.40 (7.24)	**<0.01**	**0.91 (<0.01)**	—	—	—	—	—
Diff	1.35 (1.35)	**<0.01**	**0.22 (0.01)**	**−0.20 (0.02)**	—	—	—	—
F1Time	**−0.26 (0.05)**	**<0.01**	**0.25 (0.04)**	**0.43 (<0.01)**	**−0.42 (0.01)**	—	—	—
F2Time	**−0.25 (0.06)**	**<0.01**	−0.13 (0.18)	−0.12 (0.19)	−0.02 (0.46)	**0.51 (<0.01)**	—	—
DTime	0.00 (0.06)	(0.46)	**0.39 (<0.01)**	**0.55 (<0.01)**	**−0.37 (0.01)**	**0.40 (0.03)**	**−0.57 (<0.01)**	—

*Note:* Diff, difference between expected fear and experienced fear; F1Time, expected fear over time; F2Time, experienced fear over time; DTime, difference between expected and experienced fear over time. Bold values indicate statistically significant results.

**Table 8:** Estimates and variances of expected fear, experienced fear and difference of expected and experienced fear and their intercorrelations (between brackets significances of the corresponding covariances) in situation: forest.

Estimates (variances)			Intercorrelations					
Fear estimates and time cores	Estimate	*p*	Expected fear	Experienced fear	Diff	F1Time	F2Time	DTime
Expected fear	5.24 (7.33)	**<0.01**	—	—	—	—	—	—
Experienced fear	4.20 (7.57)	**<0.01**	**0.92 (<0.01)**	—	—	—	—	—
Diff	1.11 (1.24)	**<0.01**	0.16 (0.08)	**−0.24 (0.02)**	—	—	—	—
F1Time	**−0.19 (0.04)**	**<0.01**	0.18 (0.14)	0.20 (0.12)	−0.06 (0.38)	—	—	—
F2Time	**−0.15 (0.05)**	**<0.01**	**−0.34 (0.02)**	**−0.33 (0.02)**	−0,02 (0.46)	**0.44 (0.03)**	—	—
DTime	−0.04 (0.05)	0.10	**0.53 (<0.01)**	**0.53 (<0.01)**	−0.02 (0.46)	**0.47 (0.04)**	**−0.56 (0.02)**	—

*Note*: Diff, difference between expected fear and experienced fear; DTime, difference between expected and experienced fear over time; F1Time, expected fear over time; F2Time, experienced fear over time. Bold values indicate statistically significant results.

**Table 9:** Estimates and variances of expected fear, experienced fear and difference of expected and experienced fear and their intercorrelations (between brackets significances of the corresponding covariances) in situation: first individual situation.

Estimates (variances)			Intercorrelations					
Fear estimates and time cores	Estimate	*p*	Expected fear	Experienced fear	Diff	F1Time	F2Time	DTime
Expected fear	6.43 (5.79)	**<0.01**	—	—	—	—	—	—
Experienced fear	5.09 (5.56)	**<0.01**	**0.89 (<0.01)**	—	—	—	—	—
Diff	1.44 (1.19)	**<0.01**	**0.24 (0.02)**	**−0.21 (0.04)**	—	—	—	—
F1Time	**−0.16 (0.03)**	**<0.01**	0.21 (0.08)	**0.27 (0.04)**	−0.14 (0.24)	—	—	—
F2Time	**−0.15 (0.04)**	**<0.01**	0.13 (0.23)	0.17 (0.15)	−0.10 (0.32)	**0.89 (<0.01)**	—	—
DTime	−0.00 (0.01)	0.46	0.13 (0.35)	0.17 (0.27)	−0.12 (0.38)	0.15 (0.42)	−0.30 (0.31)	—

*Note*: Diff, difference between expected fear and experienced fear; DTime, difference between expected and experienced fear over time; F1Time, expected fear over time; F2Time, experienced fear over time. Bold values indicate statistically significant results.

**Table 10:** Estimates and variances of expected fear, experienced fear and difference of expected and experienced fear and their intercorrelations (between brackets significances of the corresponding covariances) in situation: second individual situation.

Estimates (variances)			Intercorrelations					
Fear estimates and time cores	Estimate	*p*	Expected fear	Experienced fear	Diff	F1Time	F2Time	DTime
Expected fear	6.40 (5.92)	**<0.01**	—	—	—	—	—	—
Experienced fear	5.01 (6.32)	**<0.01**	**0.82 (<0.01)**	—	—	—	—	—
Diff	1.49 (2.33)	**<0.01**	**0.23 (0.01)**	**−0.36 (<0.01)**	—	—	—	—
F1Time	**−0.19 (0.05)**	**<0.01**	0.12 (0.20)	0.13 (0.19)	−0.05 (0.40)	—	—	—
F2Time	**−0.15 (0.04)**	**<0.01**	0.11 (0.26)	−0.08 (0.33)	0.30 (0.09)	**0.75 (<0.01)**	—	—
DTime	−0.04 (0.03)	0.06	0.03 (0.42)	0.28 (0.06)	**−0.45 (0.02)**	**0.54 (0.03)**	−0.13 (0.37)	—

*Note*: Diff, difference between expected fear and experienced fear; DTime, difference between expected and experienced fear over time; F1Time, expected fear over time; F2Time, experienced fear over time. Bold values indicate statistically significant results.

**Table 11:** Prediction of treatment outcome at postassessment of experienced fear and difference between expected and experienced fear.

Outcome measures	Experienced fear	Diff	F2Time	DTime	*R* ^2^
ACQ	0.18 (0.50)	0.20 (0.43)	**−0.63 (<0.01)**	0.33 (0.48)	0.70 (0.14)
BSQ	0.20 (0.43)	−0.01(0.95)	**−0.86 (<0.01)**	0.20 (0.63)	0.71 (0.07)
MI acc	0.31 (0.31)	0.06 (0.83)	−0.62 (0.10)	0.22 (0.62)	0.42 (0.66)
MI unac	0.16 (0.50)	0.04 (0.86)	**−0.81 (<0.01)**	0.47 (0.21)	**0.64 (0.04)**
PAS	0.33 (0.28)	0.11 (0.66)	**−1.05 (<0.01)**	0.39 (0.27)	**0.99 (0.05)**

*Note:* Beta scores (with significances of corresponding regression coefficients in brackets) and *R*^2^ (between brackets overall significance) are reported. MI acc, mobility inventory, subscale accompanied; MI unac, mobility inventory, subscale unaccompanied. Diff, difference between expected fear and experienced fear; F2Time, experienced fear over time; DTime, difference of expected and experienced fear over time. Note that the F2Time value for PAS is greater than one. This is an indication of multicollinearity. Bold values indicate statistically significant results.

Abbreviations: ACQ, Agoraphobic Cognitions Questionnaire; BSQ, Bodily Sensations Questionnaire; PAS, Panic and Agoraphobia Scale.

**Table 12:** Prediction of treatment outcome at follow-up assessment of experienced fear and difference between expected and experienced fear.

Outcome measures	Experienced fear	Diff	F2Time	DTime	*R* ^2^
ACQ	0.11 (0.32)	0.26 (.22)	**−0.86 (0.01)**	0.29 (0.32)	0.69 (0.07)
BSQ	0.23 (0.33)	0.05 (.79)	**−0.82 (<0.01)**	0.17 (0.64)	**0.76 (0.03)**
MI acc	0.38 (0.24)	−0.05 (.84)	−0.64 (0.08)	0.01 (0.97)	0.39 (0.59)
MI unac	0.30 (0.18)	0.15 (.47)	**−0.80 (0.04)**	0.41 (0.21)	0.73 (0.14)
PAS	0.37 (0.09)	0.05 (.79)	**−0.98 (<0.01)**	0.25 (0.34)	**0.99 (<0.01)**

*Note:* Beta scores (with significances of corresponding regression coefficients in brackets) and *R*^2^ (between brackets overall significance) are reported. MI acc, mobility inventory, subscale accompanied; MI unac, mobility inventory, subscale unaccompanied. Diff, difference between expected fear and experienced fear; F2Time, experienced fear over time; DTime, difference of expected and experienced fear over time. Bold values indicate statistically significant results.

Abbreviations: ACQ, Agoraphobic Cognitions Questionnaire; BSQ, Bodily Sensations Questionnaire; PAS, Panic and Agoraphobia Scale.

## Data Availability

The data that support the findings of this study are available from the corresponding author upon reasonable request.
